# Agreement between Fetal Brain Ultrasonography and Magnetic Resonance Imaging in the Measurements of the Corpus Callosum and Transverse Cerebellar Diameter

**DOI:** 10.3390/diagnostics14040366

**Published:** 2024-02-07

**Authors:** Shai Bookstein, Noy Nachmias, Eldad Katorza

**Affiliations:** 1 Antenatal Diagnostic Unit, Department of Obstetrics and Gynecology, Chaim Sheba Medical Center, Tel-Hashomer, Derech Sheba 2, Ramat Gan 5262000, Israel; bookstein1@mail.tau.ac.il (S.B.); noy.na87@gmail.com (N.N.); 2 Faculty of Medicine, Tel Aviv University, Klatchkin 35, Tel Aviv 6139001, Israel; 3 Gertner Institute for Epidemiology & Health Policy Research, Chaim Sheba Medical Center, Tel-Hashomer, Derech Sheba 2, Ramat Gan 5262000, Israel; 4 Arrow Program for Medical Research Education, Chaim Sheba Medical Center, Tel-Hashomer, Derech Sheba 2, Ramat Gan 5262000, Israel

**Keywords:** fetal brain, MRI, corpus callosum, cerebellum, neurodevelopment

## Abstract

As the use of magnetic resonance imaging of the fetal brain has evolved, the need to understand its efficiency in the biometry of the fetal brain has broadened. This study aimed to assess the level of agreement and correlation between the two cardinal imaging methods of fetal neuroimaging, ultrasonography (US) and magnetic resonance imaging (MRI), by measuring the corpus callosum (CC) and transverse cerebellar diameter (TCD) in terms of length and percentile. Measurements of CC and TCD length and percentile were documented over a 7-year span in a tertiary referral medical center. All US and MRI examinations were performed in the customary planes and subcategorized by valid reference charts. Exclusion and inclusion criteria were set before the collection and processing of the data. A total of 156 fetuses out of 483 were included in the study. A positive, strong correlation and agreement were found (r = 0.78; ICC = 0.76) between US and MRI in TCD measurements. For CC length measurement, a moderate correlation and moderate agreement (r = 0.51; ICC = 0.49) between US and MRI was observed. TCD and CC percentiles had lower levels of correlation and agreement compared with the length variables. Our study indicates good agreement between MRI and US in the assessment of TCD measurement as a part of antenatal neuroimaging. Furthermore, while the two techniques are not always compatible, they are complementary methods.

## 1. Introduction

Over the last two decades, the use of magnetic resonance imaging (MRI) of the fetal brain has evolved substantially [1]. Although MRI is becoming more available, ultrasound (US) examination is still the method of choice; hence, MRI is used only when there are suspected abnormalities in fetal development or difficulties in imaging during ultrasound screening [2]. MRI is being used to confirm or exclude suspected abnormalities from the US and might assist in the detection of new abnormalities [3].

One of the cardinal evaluations in fetal development is the fetal brain. As a part of this screening, the length of the corpus callosum (CC) and transverse cerebellar diameter (TCD) are being measured [4].

The CC and cerebellum are both important in the assessment of prenatal screening, as abnormalities in their anatomical structure can lead to far-reaching consequences in the neurodevelopmental and general development of the fetus [4,5].

TCD is an obligatory measurement in fetal biometry since it is used to detect fetal growth restriction [6], and it is associated with chromosomal abnormalities, such as trisomy 18, and other malformations, such as Dandy–Walker malformation [7,8].

Furthermore, the CC is an interhemispheric structure that constitutes the largest white matter tract. Identifying the CC in the fetal US might be difficult, even in a healthy fetus [3]; thus, the need for MRI in the diagnosis of malformations in the CC is substantial. CC dysgenesis is the second most important indication for fetal MRI, and the prognosis is often uncertain [9], but while malformations of the CC are uncommon, callosal dysgenesis correlates with a high risk for developmental delay [1].

**Figure 1:**
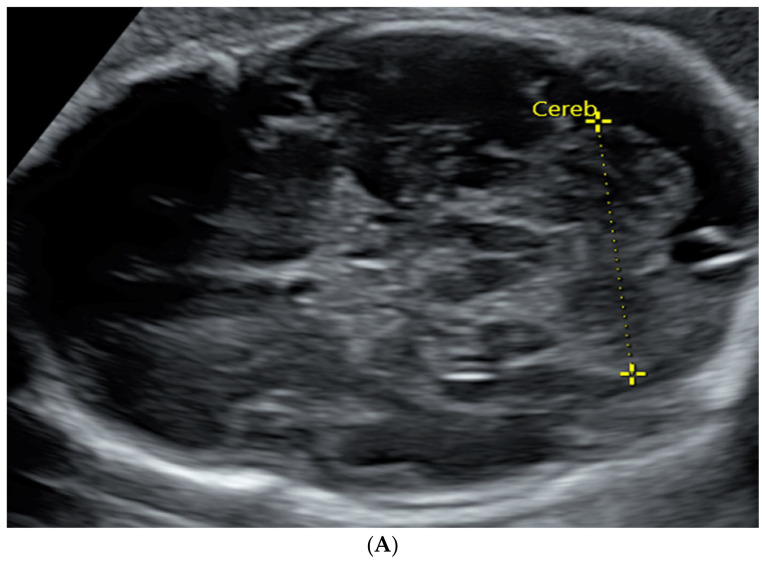

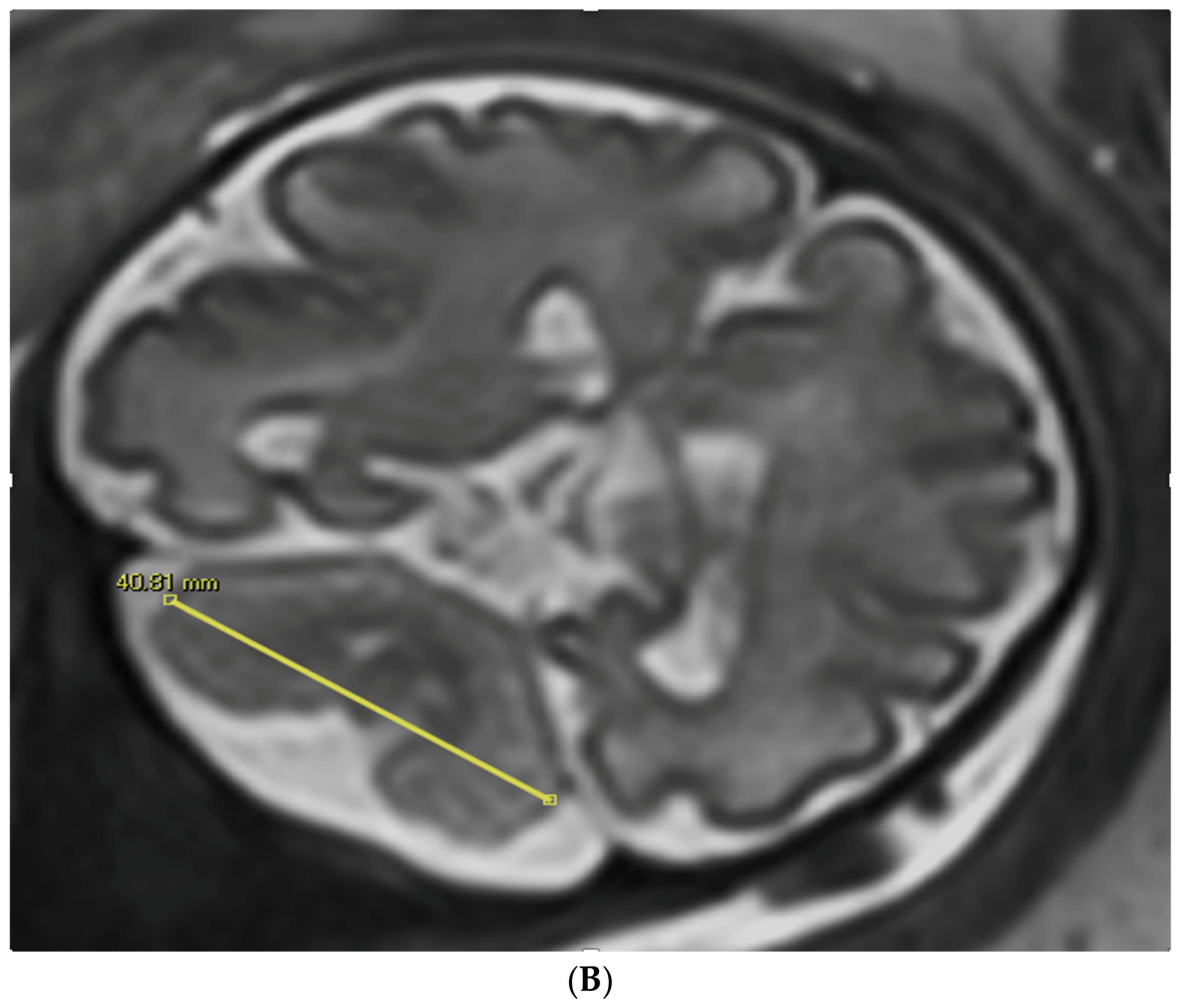
TCD measured in an image by trans-abdominal axial US scan at 31 weeks of gestation (**A**) and by coronal MRI scan at 32 weeks of gestation (**B**).

**Figure 2:**
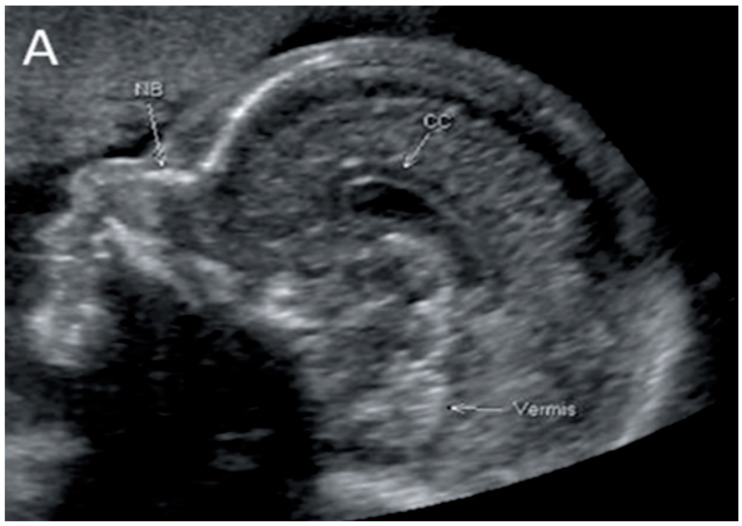

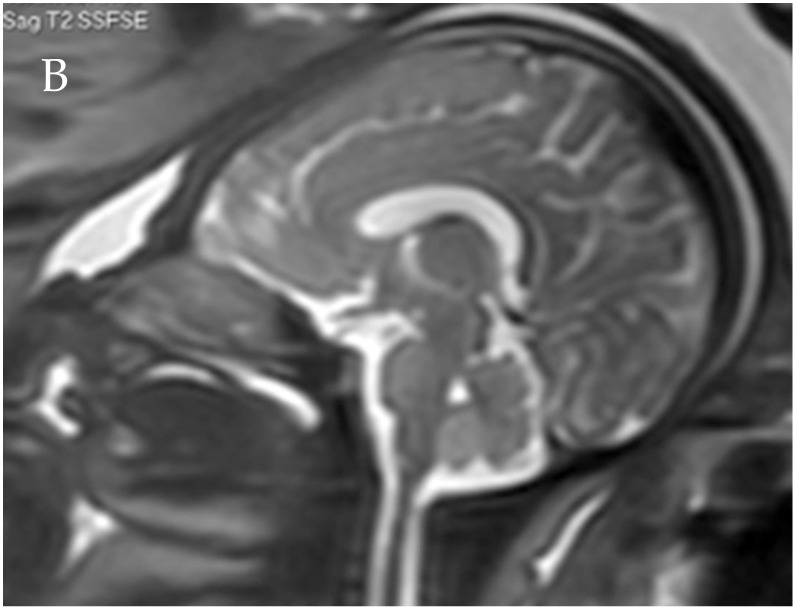
Corpus callosum as captured by trans-abdominal mid-sagittal US scan at 23 weeks of gestation (**A**) and by mid-sagittal MRI scan at 33 weeks of gestation (**B**).

TCD and CC length are both visualized by US and MRI (Figure 1 and Figure 2).

While US is more accessible, cost-effective, and examines the fetus in real time, MRI has better visualization of the white matter and other brain structures, independent of fetus presentation, and is hence considered more reliable [10,11]. MRI significantly improves the accuracy and confidence of fetal brain anomaly diagnosis [12].

We hypothesize that there will be a good agreement in the biometry measurements and a good agreement in the percentile measurements, but the magnitude of agreement will be smaller than in the biometry due to differences in the methods of creating the percentile tables between the imaging modalities. Moreover, Perlman et al. showed an excellent agreement between the two imaging modalities as to the diagnosis of fetal ventriculomegaly [13].

The aim of our study was to evaluate the agreement and correlation between US and MRI in the measurements of TCD and CC length in the customary sectional planes for each technique in prenatal diagnosis.

Many studies have been conducted to construct reference charts of CC length in either imaging modality, which emphasizes the clinical need to have the ability to interpret the data from the imaging to make clinical decisions and improve parental counseling. The studies differ from each other in some quality measures, such as inclusion criteria, and these differences might affect their results and the concordance between them [14].

Moreover, another study by Resta et al. showed the significance of complementary MRI in cases that are uncertain by routine US or when more accurate anatomical detail is required. MRI provides additional information about corpus callosum malformations in patients with ventriculomegaly suspected by US [15]. Accordingly, fetal MRI has an important role in the detection of brain anomalies, and the ability to utilize it as a complementary imaging method to US in brain biometry is beneficial to both clinicians and patients.

## 2. Materials and Methods

### 2.1. Study Design

This study is a retrospective study performed in a single tertiary referral medical center, expert in fetal neuroimaging, during a 7-year period (2012–2018).

Inclusion criteria were singleton pregnancy, ultrasound dating performed in the first trimester of pregnancy, MRI examination without any pathological findings, CMV seroconversion without any witness of neurological damage, absence of chromosomal findings in genetic tests or findings without known meaning, ventricular asymmetry with a difference up to 4 mm, the time difference between the US and the MRI is shorter than two weeks, mild cystic findings (such as CPC), family history of neurodevelopmental problems.

Exclusion criteria were as follows: multifetal pregnancy, bad quality of MRI examination, existing neurological defects (neural crest defects, agenesis, or hypoplasia of corpus callosum, abnormal posterior fossa, and cerebellar defects), intraventricular hemorrhage, cortical defects, and lissencephaly.

The mean and median gestational age when performing the MRI was 32.7 ± 1.64 and 32.71 weeks, respectively (range 26–38 weeks). Mean and median gestational ages when performing the US screening were 32.1 ± 1.89 and 32.07 weeks, respectively (range 24–37 weeks). US screening and MRI were both performed for each fetus with a time difference that did not exceed two weeks, so the difference in the biometry will less likely be attributed to a significant change in the true measures and to the differences similar between patients. In accordance with this limitation, the mean time differences and standard deviation (SD) between US and MRI was 4.34 ± 6.79 days.

### 2.2. MR Imaging Scans

Fetal brain MRI was performed using a 1.5T system (Optima 1.5T; GE Healthcare Ultrasound, Milwaukee, WI, USA). Single-shot fast spin-echo T2-weighted sequences in 3 orthogonal planes were performed according to the listed parameters: section thickness of 3–4 mm; no gap; flexible coil (8-channel cardiac coil); matrix 320 × 224, echo time (TE) of 90 ms; and repetition time (TR) of 1298 ms. The field of view (FOV) was determined by the size of the fetal head: 24 cm for the smaller fetuses and for the larger fetuses 30 cm. T1 fast-spoiled gradient-echo sequences were performed only in the axial plane with a larger FOV of 400 mm, with section thickness 4 mm, gap 0.5 mm, TR 160 ms, and TE 2.3 ms.

The length of corpus callosum was measured from the genu to the posterior aspect of the splenium in the same manner as measured by Garel [16], in the construction of the reference charts that were used to subcategorize the measurements to the specific percentile in this study.

All MRI measurements of the corpus callosum (CC) and transverse cerebellar diameter (TCD) were subcategorized according to reference charts published by B. Tilea et al. in 2009, which were based on a large cohort of 589 fetuses and enabled percentile estimation of 5th and 95th percentiles [17].

### 2.3. Ultrasound Scans

Ultrasound examination of the fetal central nervous system was performed as described in the International Society of Ultrasound in Obstetrics and Gynecology guidelines [4] and included the shape of the fetal head (measured by biparietal diameter, BPD, and head circumference, HC), lateral ventricles, and choroid plexus, corpus callosum, cerebellum, cisterna magna, thalami, and cavum septi pellucidi (CSP) using transventricular, transcerebellar and transthalamic planes. Transverse and longitudinal sections of the fetal spine were obtained to rule out spine malformations, such as spina bifida. A multiplanar approach (4-coronal and 3-sagittal planes) was performed to evaluate brain selectivity precisely. All examinations were performed using Voluson E6 or E8 Expert ultrasound machines (GE Healthcare, Kretz ultrasound, Zipf, Austria) with 4–8 MHz transabdominal transducer or transvaginal transducer of 5–9 or 6–12 MHz. By default, measurements of the corpus callosum length and thickness were performed using the transabdominal route, except in cases where fetal position prevented or limited the measurement ability by the transabdominal route, and therefore, transvaginal transducer and route were used.

All ultrasonographic measurements of the corpus callosum were subcategorized according to the corpus callosum reference charts published by Cignini et al. in 2014, which were based on the largest cohort known so far of 2950 fetuses [5].

All ultrasonographic measurements of the transverse cerebellar diameter (TCD) were subcategorized according to reference charts of transverse cerebellar diameter published by Hill et al. in 1990, which were based on a cohort of 675 women with singleton pregnancy and normal gestation [18].

### 2.4. Statistical Analysis

Based on previous work by Gafner et al., which showed that there is no statistically significant difference in the TCD and in the length of CC between the different sexes [19], we performed the analysis on all cases without dividing into groups based on fetal sex.

Categorical variables were reported as numbers and percentages. Continuous variables were evaluated for normal distribution using histograms and Q-Q plots and are reported as mean and standard deviation.

A paired samples *t*-test was used to compare the methods. The Pearson correlation coefficient was used to evaluate the correlation between the two methods.

Interclass correlation coefficient (ICC) was used to evaluate the absolute agreement between the two methods. The agreement was considered slight when ICC ≤ 0.2, fair when 0.21 ≤ ICC ≤ 0.4, moderate when 0.41 ≤ ICC ≤ 0.6, good when 0.61 ≤ ICC ≤ 0.8, and very good when ICC > 0.8. Bland and Altman plot was used to describe the agreement [20].

All statistical tests were two-sided, with a significance level of 0.05 considered significant. All measurements were conducted using IBM SPSS statistics, version 24 (IBM Cooperation, Armonk, NY, USA).

The study was approved by the local institutional review board of the Sheba Medical Center.

## 3. Results

### 3.1. Demographic and Clinical Characteristics of the Study Population

**Figure 3:**
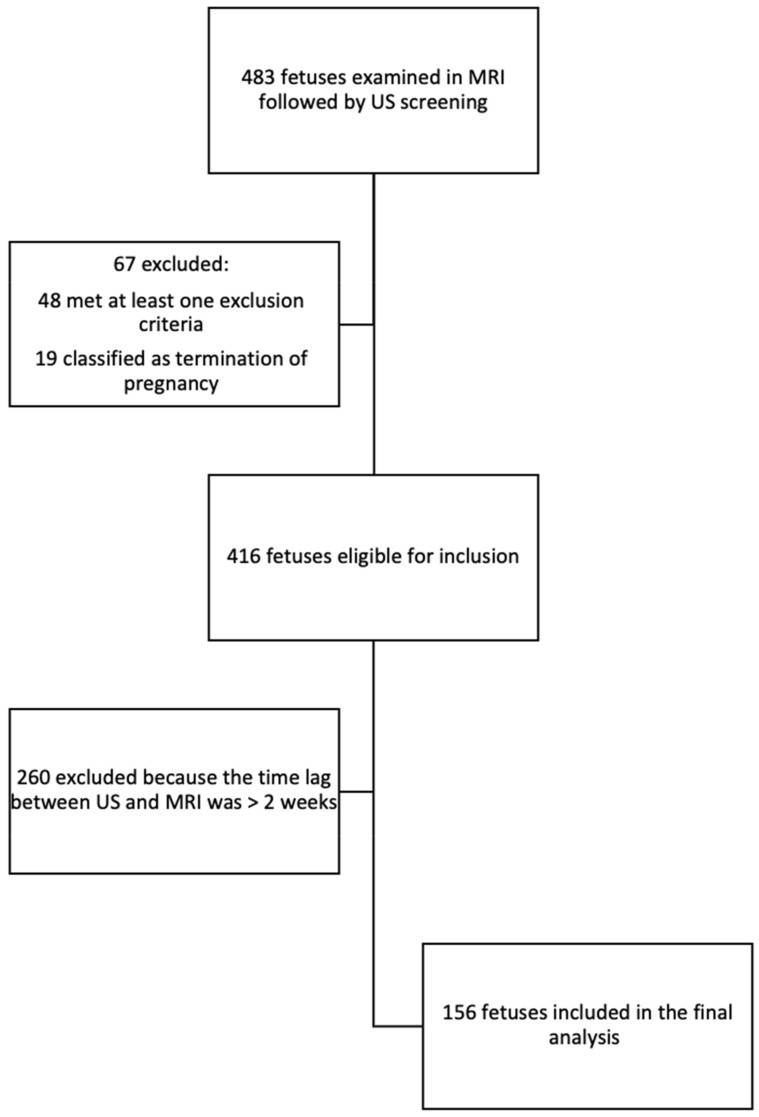
Flow diagram of inclusion in the study and analysis.

A total of 483 women underwent an MRI examination followed by an ultrasound examination, of whom 67 were excluded, 19 due to termination of pregnancy, and 48 met at least one exclusion criterion. After the preliminary selection, 260 were excluded from the study because of a time lag that exceeded 2 weeks between the US and MRI examinations. Eventually, 156 fetuses were included in this study (Figure 3). 

As listed in Table 1, of the 156 fetuses that were included in the study, 58 were female (37.2%), and 98 (62.8%) were male.

Most fetuses were at head presentation (140, 89.7%) during MRI examination, while only 13 (8.1%) fetuses were at breech presentation, and 3 (1.9%) were at transverse presentation.

The most common indication for MRI examination was ventricular asymmetry in 94 out of 156 fetuses, which included a difference not larger than 4 mm, as stated in the inclusion criteria. Furthermore, 7.7% had cystic lesions, which included black pouch cysts and choroid plexus cysts (CPCs). Another 5.1% had undetermined genetic findings, which included minimal deficit in chromosome 3, duplication of chromosome 1, reciprocal translocation of 13 and 14 chromosomes, family history of polydactyly, and family history of developmental delay and microcephaly. A further 5.1% had CMV seroconversion during pregnancy, and 5.8% had a history of CNS illness, meaning that they had a previous termination of pregnancy due to intracranial hemorrhage, defects in sulcus formation, and cortical damage. Furthermore, 19.9% had other suspected CNS illnesses such as suspicion of spina bifida during US screening, suspicion of large or small head circumference in US examination, scalp edema, and a narrow cavum septum pellucidum (CSP). Finally, 3.8% had miscellaneous indications for fetal MRI, which included club foot, horseshoe kidney, pelvic cyst and single umbilical artery, hypoplastic heart, suspicion for polyhydramnios, and suspicion for face formation defect (Table 1).

### 3.2. Correlation Assessment of MRI and US Measurements

Correlation was calculated to explore the relationship between the measurements by the different imaging modalities. Pearson correlation (r) was used to determine whether there is an association between the measurement of the length of the corpus callosum, transverse cerebellar diameter, and percentiles of CC and TCD (Table 2).

For most variables (TCD percentile, CC length, and percentile), a moderate correlation was found (r > 0.3). However, a positive, strong correlation (r = 0.78) was found in TCD measurements with the smallest mean difference (0.02 ± 2.85 mm) between the two modalities (Table 2).

### 3.3. Agreement Estimation between MRI and Ultrasonography

Agreement was calculated to explore whether the measurements from the different imaging modalities were comparable. A fair agreement was found in the evaluation of the corpus callosum percentile (ICC = 0.36), while a moderate agreement was found in the measurements of corpus callosum length (ICC = 0.49, Table 3).

In the evaluation of transverse cerebellar diameter and TCD percentile, a moderate agreement (ICC = 0.44) was found in the cerebellum percentile, while a good agreement was found in the evaluation of TCD (ICC = 0.76; Table 3).

In the comparison between the agreement of length and percentile, the agreement between the length of corpus callosum and transverse cerebellar diameter was better than the agreement between the percentile for each variable relatively. There was a moderate agreement in CC length (ICC = 0.49) compared with a fair agreement in the CC percentile (ICC = 0.36) and a good agreement in TCD length (ICC = 0.76) compared with a moderate agreement in TCD percentile (ICC = 0.44) (Table 3).

**Figure 4:**
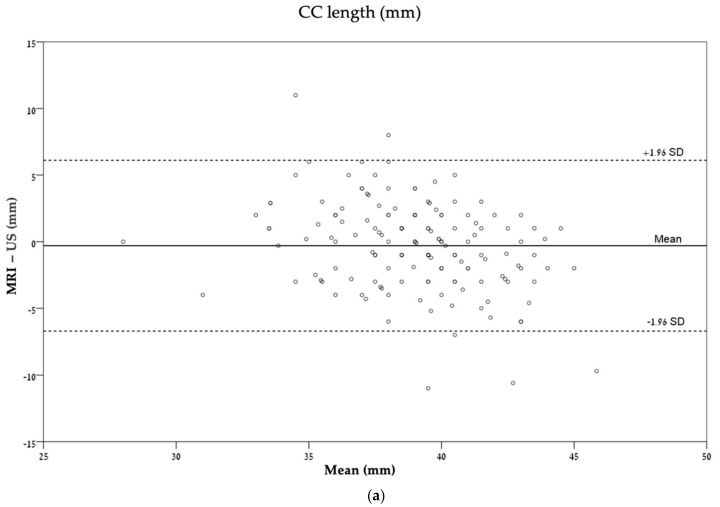

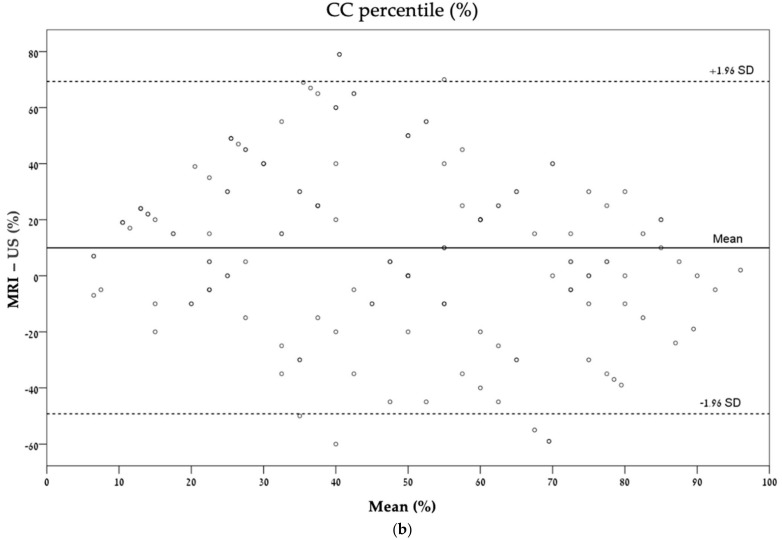

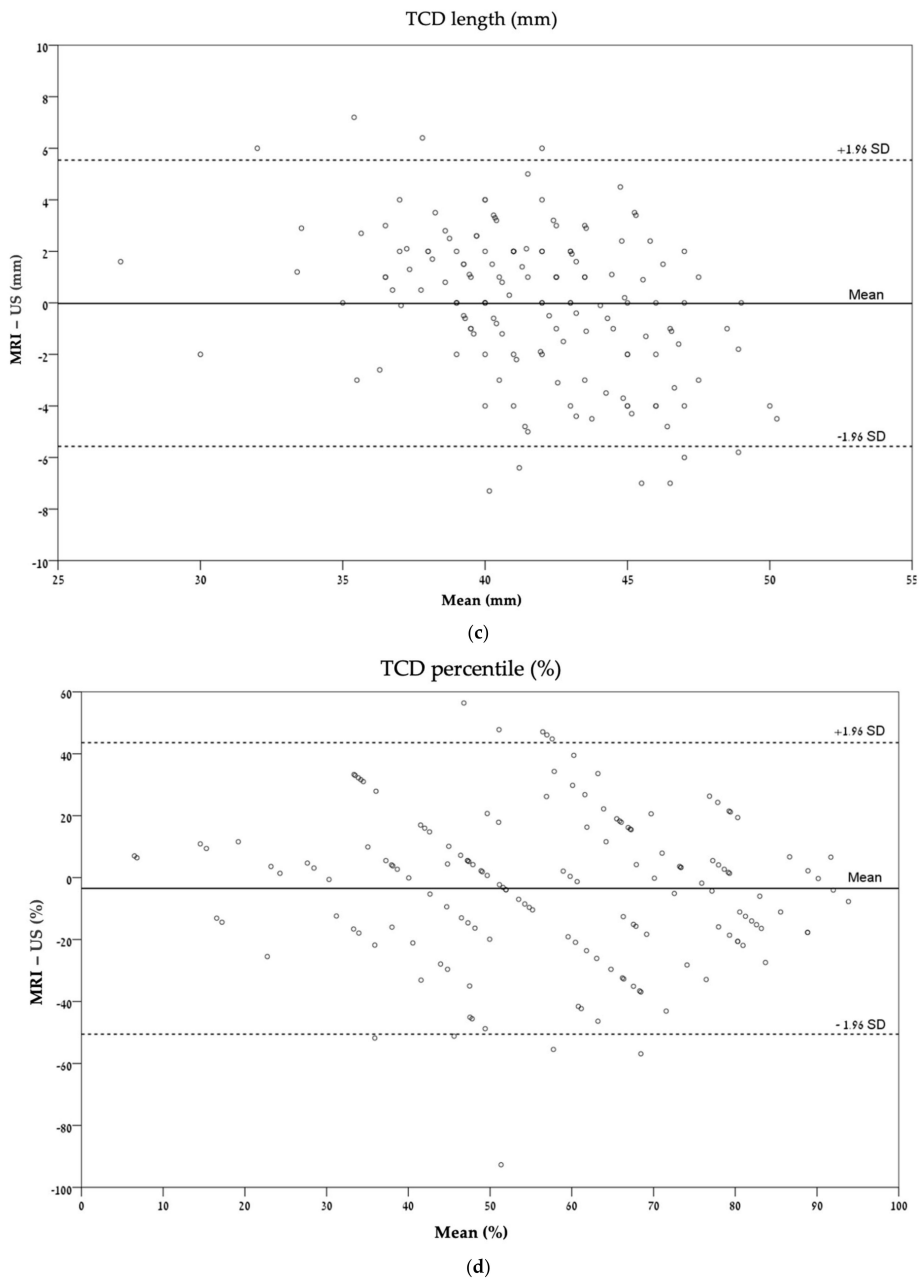
Bland–Altman plots describing the agreement between ultrasonography and MRI for each measurement: (**a**) CC length (mm); (**b**) CC percentile (%); (**c**) TCD (mm); (**d**) TCD percentile (%). CC, corpus callosum; TCD, transverse cerebellar diameter.

These agreements are also described graphically by Bland–Altman plots (Figure 4).

In accordance with Table 3, which presents the ICC (interclass correlation), the plots demonstrated a wide distribution of dots around the mean, with no particular pattern except for Figure 4c, which describes the agreement of transverse cerebellar diameter (mm).

## 4. Discussion

### 4.1. Key Results

In this study, we examined whether there is an agreement between the two main modalities in prenatal neuroimaging: ultrasonography and MRI.

We found a good agreement and a positive, strong correlation between the two modalities in the measurement of TCD. In addition, when comparing length to percentile variables, the agreement in the length of corpus callosum and TCD was higher than the percentile for each of them.

### 4.2. Interpretation and Limitations of the Study

The importance of assessing the agreement between the two modalities is crucial for clinical decision making since MR imaging is becoming more common in prenatal diagnoses [1,2]. Thus, it is necessary to understand whether the evaluation of the fetal brain is comparable between US, the more routinely used modality, and MRI.

According to our study, the level of agreement was low for most variables. This can be explained by the fact that MRI and US are two different modalities, and as such, they have different imaging techniques, and this difference might affect the visualization and measurement of the fetal brain structures.

While US allows the measurement of the fetal brain in real time and has fewer artifacts from the movement of the fetus, it is still operator dependent, and hence, the measurement might not be reliable, as its consistency is dependent on the specific operator in each clinic. However, MRI can visualize the fetal brain in a higher resolution and help assess other brain anomalies that might be present with an anomaly in the corpus callosum, but it is less accessible, less frequently used, and does not allow for observation of the fetal brain in real time.

In addition, US, while a low-cost and accessible method of choice, is still affected by maternal obesity, fetal presentation, and oligohydramnios [10]. Hence, in cases in which US might not allow an accurate measurement and visualization, with the possible need to use MRI, it is necessary to have the ability to interpret the results in comparison to the population.

Nevertheless, our study did find a close agreement between TCD measurements. Our results are in accordance with the results of Tilea et al. [17] and can be explained by the presumption that TCD measurements are well established and examined as a part of routine screening [4].

In comparison to corpus callosum length, which is not ordinarily screened [4], it is plausible to assume that TCD measurements will be more accurate and, hence, have a stronger correlation and better agreement.

It is important to mention that the retrospective nature of this study imposes several limitations. While each ultrasonography examination and MRI were performed by an expert in the field, they were performed by different operators, and it might affect the results. In addition, although we included only US screening and MRI with a time difference that was no longer than 2 weeks, during this time, the fetus continued to evolve and grow. This can lead to differences in measurements. Moreover, the need to meet certain eligibility criteria might affect the external validity and generalizability of the study, as only a selected population from the general target population was included in the study.

Our study is in line with other studies that have attempted to examine whether there is an agreement between MRI and US as a part of fetal neuroimaging [10,11,13,20]. Currently, MRI is believed to be more accurate due to its many advantages, such as a higher contrast resolution and its ability to detect abnormalities that can be overlooked in US [11,20]. Furthermore, MRI can influence decisions regarding pregnancy management and outcome [21,22].

However, according to our results, a good agreement was found only in TCD length. This may imply that US and MRI are not always compatible but complementary; the combination of the two can lead to thorough fetal brain imaging and better decision making.

When comparing percentile variables to length variables (TCD length to TCD percentile and CC length to CC percentile), we observed that the level of agreement was relatively lower than the level of agreement for length variables. This can be explained by the fact that each length variable was subcategorized according to different reference charts: different sonography reference charts for CC and TCD [5,18] and another reference chart for the MRI data [17]. The use of multiple reference charts can lead to a variance in percentile estimation. In addition, the study population in which the MRI reference charts were developed is different than the population of the ultrasonography studies. The study by Tilea [17] et al. was an observational study that included only fetuses that had a specific indication to undergo MRI examination, whereas the US study included the measurement of all women who requested a fetal biometric evaluation (except in cases in which the fetus had a high risk of CNS anomaly). Due to the fact that the populations are different in their characteristics, it is reasonable to assume that there will be a difference in the percentile evaluation. Additionally, the MRI chart is constructed based on 589 fetuses, while the US chart is based on 2950 fetuses. The relatively small sample size for the MRI study might affect the external validity of the reference chart, as a smaller sample might not represent the study population accurately. Hence, it might affect the results of our study regarding the agreement of the percentiles of the biometry measures. Thus, our study emphasizes the complexity of using different percentile reference charts for different modalities and different measurements. Our study raises questions regarding the applicability of using different reference charts for different modalities. The difference in percentile, which is larger than the difference in length, might stem from the difference in the populations on which the reference charts were based.

## 5. Conclusions

US and MRI are both cardinal methods for fetal brain evaluation. While US is more routinely used for screening, when there is a suspected finding, the patient might be recommended to undergo MRI evaluation to examine the findings and evaluate the suspicion of an anomaly. The combination of the two contributes to a complete assessment. According to our study, it was indicated that the measurement of transverse cerebellar diameter, as accepted in US and MRI, is correlative and has a good agreement. Moreover, our study emphasizes the need to create comparable reference percentile charts for the different modalities. Further research is needed to explore the correlation of the measurements with the different modalities using postpartum measurements.

## Figures and Tables

**Table 1:** Descriptive data of the fetuses included in the study and their indication for fetal brain MRI.

	Number	Percent (%)
Gender		
Female	58	37.2
Male	98	62.8
Presentation		
Head	140	89.7
Breech	13	8.1
Transverse	3	1.9
Indication		
Ventricular asymmetry	94	60.3
Cystic lesions	12	7.7
Genetic findings	8	5.1
CMV seroconversion	8	5.1
History of CNS illness	9	5.8
Other CNS findings	19	12.2
Miscellaneous	6	3.8

Describes data of all 156 fetuses included in the study. CMV, cytomegalovirus; CNS, central nervous system.

**Table 2:** Correlation between ultrasonography and MRI data in the evaluation of corpus callosum and cerebellum length and percentile.

	Ultrasound Mean ± SD	MRI Mean ± SD	Absolute Differences in Measurements Mean ± SD	Significance	Correlation (r)	*p*-Value
CC length (mm)	39.15 ± 3.67	38.85 ± 2.76	−0.29 ± 3.28	0.27	0.51	<0.001
CC percentile (%)	42.35 ± 30.22	52.31 ± 24.31	9.96 ± 30.35	<0.001	0.39	<0.001
TCD (mm)	41.70 ± 4.56	41.68 ± 3.53	−0.02 ± 2.85	0.92	0.78	<0.001
TCD percentile (%)	58.76 ± 23.74	55.30 ± 21.82	−3.46 ± 24.03	0.07	0.45	<0.001

Correlation between MRI and US measurements of CC and transverse cerebellar diameter. The difference between MRI and US in length (mm) and percentile was calculated as the absolute value of MRI minus US. Pearson correlation coefficient is indicated by r. SD, standard deviation; CC, corpus callosum; TCD, transverse cerebellar diameter.

**Table 3:** Agreement between MRI and US.

	ICC	95% CI	Significance
CC length (mm)	0.49	0.36–0.60	<0.001
CC percentile (%)	0.36	0.21–0.49	<0.001
TCD (mm)	0.76	0.68–0.82	<0.001
TCD percentile (%)	0.44	0.31–0.56	<0.001

Representation of the agreement between measurements of corpus callosum and cerebellum by the two modalities of MRI and US. ICC, interclass correlation coefficient; CC, corpus callosum; TCD, transverse cerebellar diameter.

## Data Availability

The data presented in this study are available on request from the corresponding author. The data are not publicly available due to privacy matters.
